# Cancer Screening and Cancer Treatment in Kidney Transplant Recipients

**DOI:** 10.34067/KID.0000000000000545

**Published:** 2024-10-31

**Authors:** Miguel Bigotte Vieira, Hiroyuki Arai, Carla Nicolau, Naoka Murakami

**Affiliations:** 1 Nephrology Department, Hospital Curry Cabral, Unidade Local de Saúde São José, Lisbon, Portugal; 2 NOVA Medical School, Universidade NOVA de Lisboa, Lisbon, Portugal; 3 Department of Nephrology, Kyoto University Graduate School of Medicine, Kyoto, Japan; 4 Division of Renal Medicine, Brigham and Women's Hospital, Boston, Massachusetts; 5 Harvard Medical School, Boston, Massachusetts

**Keywords:** cancer, kidney transplantation, organ transplant, renal transplantation, transplant outcomes, transplantation

## Abstract

As the population ages and post-transplant survival improves, pretransplant and post-transplant malignancy are becoming increasingly common. In addition, rapid advances in cancer therapies and improving outcomes prompt us to rethink pretransplant cancer-free wait time and screening strategies. Although kidney transplant recipients (KTRs) are at higher risk of developing cancer, epidemiological data on how to best screen and treat cancers in KTRs are incomplete. Thus, current recommendations are still largely on the basis of studies in the general population, and their validity in KTRs is uncertain. Kidney transplant candidates without prior cancer should be evaluated for latent malignancies even in the absence of symptoms. Conversely, individuals with a history of malignancy require thorough monitoring to detect potential recurrences or *de novo* malignancies. When treating KTRs with cancer, reducing immunosuppression can enhance antitumor immunity, yet this also increases the risk of graft rejection. Optimal treatment and immunosuppression management remains undefined. As the emergence of novel cancer therapies adds complexity to this challenge, individualized risk-benefit assessment is crucial. In this review, we discuss up-to-date data on pretransplant screening and cancer-free wait time, as well as post-transplant cancer screening, prevention strategies, and treatment, including novel therapies such as immune checkpoint inhibitors and chimeric antigen receptor T-cell therapies.

## Introduction

Cancer is a leading cause of morbidity and mortality in kidney transplant recipients (KTRs) and patients with ESKD. These patients present a two- to four-fold increased risk of malignancy compared with the age-matched and sex-matched population.^1–3^ The increase in risk is cancer specific, being higher for Kaposi sarcoma (incidence up to 500 times more frequent) and nonmelanoma skin cancer (NMSC, up to 40 times), as well as for virus-related cancers, such as post-transplant lymphoproliferative disorder (PTLD).^1,4^ In KTRs, their prognosis is worse than in the general population in all cancer types and management is challenging. The disease course is frequently more aggressive, and therapeutic options are limited because of patients presenting multiple comorbidities and the risk of rejection and graft loss after reduction in immunosuppression.^5,6^ Preventive measures are paramount. Screening allows early detection of malignancy and might increase the chance of curative treatment in some types of cancer, reducing morbidity and mortality.^7^ Here, we review available screening guidelines for transplant candidates and KTR, cancer general management, and new therapies aimed at improving outcomes in KTRs.

## Cancer Screening and Cancer-Free Wait Time for Transplant Candidates

Although CKD increases malignancy risk, screening uptake is lower than in patients without CKD.^8^ Candidates without a history of malignancy should be evaluated for asymptomatic malignancies. Kidney Disease Improving Global Outcomes (KDIGO) guidelines recommend that kidney transplant candidates undergo routine cancer screening, as per local guidelines for the general population.^9^ Cancers of the urinary tract system are associated with ESKD. KDIGO recommends ultrasonography in candidates at increased risk for renal cell carcinoma (RCC) and suggests cystoscopy in candidates at increased risk of bladder carcinoma.^9^ Screening for hepatocellular carcinoma in candidates with cirrhosis before transplantation is also recommended.^9^

As the ESKD population ages and cancer treatments improve, more patients with a history of cancer are evaluated for solid organ transplantation (SOT).^10,11^ Active malignancy (excluding NMSC) constitutes an absolute contraindication to kidney transplantation. The likelihood of recurrence varies depending on tumor type. The benefits of transplantation should be weighed up against the risk of tumor recurrence because immunosuppression dose and regimen increase the risk of cancer recurrence and potentially affect post-transplant survival.^12^ The timing of SOT in patients with a history of malignancy hinges on various factors, including stage at diagnosis, time from treatment, treatment response, required organ, and level of immunosuppression. Historically, most clinical guidelines recommended a recurrence-free waiting period of 2–5 years before candidate listing. However, this interval should be individualized as recent population-based studies show lower cancer recurrence rates than initially reported.^13^ Despite risks, overall patient survival with transplantation may exceed expectations, with therapy advancements potentially improving recurrence outcomes. The American Society of Transplantation (AST) and the International Transplant Skin Cancer Collaborative published a thorough consensus expert opinion statement with waiting period recommendations for various cutaneous, hematological, and solid organ malignancies.^10,12,13^ In Table 1, we outline recommended wait time for SOT candidates with a history of the main cancer types.

**Table 1:** Recommended wait time for solid organ transplantation candidates with a history of breast, lung, colon, rectal, gynecological, renal cell carcinoma, bladder, prostate, or skin cancer

Cancer	Risk/Stage/Type	5-yr Disease-Specific Survival	Time Interval to Transplant	Additional Considerations
Breast	Low risk • DCIS • Stage 1	97%–99%	No wait time necessary^a^	Hormone receptor negative disease may have a slightly higher risk of recurrence in the first 2–3 yr
	Intermediate risk • Stage 2	90%–99%	1–2 yr NED^a^	Hormone receptor negative disease may have a slightly higher risk of recurrence in the first 2–3 yr
	High risk • Stage 3	66%–97%	3–5 yr NED^a^	Hormone receptor negative disease may have a slightly higher risk of recurrence in the first 2–3 yr Inflammatory breast cancer likely has a higher risk of recurrence and worse survival
	Prohibitive risk • Stage 4	32%–38%	Not a SOT candidate	
Lung	1			
	T1aN0	92%	≥3 yr PET-CT; consider biopsy post-SBRT^b^	
	T1bN0	83%	≥3 yr PET-CT; consider biopsy post-SBRT^b^	
	T1cN0	77%	3–5 yr PET-CT; consider biopsy post-SBRT^b^	5-yr recurrence-free survival is safest
	1B			
	T2aN0	68%	5 yr PET-CT^b^	
	2A			
	T2bN0	60%	5 yr PET-CT^b^	
	2B			
	T3N0	53%	5 yr PET-CT^b^	
	3A	36	5 yr PET-CT^b^	Special caution with N2 disease
	3B	26	N/A	Not an SOT candidate
	3C	13	N/A	Not an SOT candidate
	4A	10	N/A	Not an SOT candidate
	4B	0	N/A	Not an SOT candidate
Colon	Low risk • Stage 1 (T1 or T2, N0, M0)	91%	1 year	Low-risk features MSI without BRAF mutation High-risk features LVI or PNI Mucinous or Signet Histology Poorly differentiated histology Bowel obstruction Tumor perforation <12 lymph nodes examined *Tumor deposits considered as N+ disease *Consider chemotherapy before transplantation for high-risk stage 2 disease *Patients with stage 3 disease should complete chemotherapy
	Low intermediate risk • Stage 2 (T3, N0, M0)	72%	2 yr, consider longer if high-risk features present	
	High intermediate risk • Stage 2 (T4, N0, M0) • Stage 3 (any T, N+, M0)		3 yr, 5 yr if high-risk features present	
	High risk • Stage 4 (any T, any N, M+)	13%	5 yr NED	SOT not recommended before 5 yr; see special consideration regarding resectable CRC metastasis
Rectal	Low risk • Stage 1 (T1 or T2, N0, M0) • Full oncologic resection	85%–88%	1 yr, consider 2 yr if high-risk features present	Low-risk features MSI without BRAF mutation Upper 1/3 rectum or rectosigmoid High-risk features LVI or PNI Mucinous or Signet Histology Poorly differentiated histology Bowel obstruction Tumor perforation <12 lymph nodes examined Lower 1/3 of rectum Incomplete mesorectal excision *Tumor deposits considered as N+ disease *Patients with stages 2 and 3 disease should complete trimodality treatment (chemoradiotherapy, surgery, and chemotherapy) unless elimination of one of these is deemed appropriate after multidisciplinary discussion *For patients who have undergone preoperative radiotherapy, response to treatment is highly prognostic
	Low intermediate risk • Stage 1 (T1, N0, M0) • Local excision	78%–88%	2 yr	
	High intermediate risk • Stage 2 (T3 or T4, N0, M0) • Stage 3 (any T, N+, M0)	70%	3 yr, 5 yr if high-risk features present	
	High risk • Stage 4 (any T, any N, M+)	14%	5 yr NED	SOT not recommended before 5 yr; see special consideration regarding resectable CRC metastasis
Gynecological cancer	Low risk • Stage 1A/1B, grade 1–2 endometrial cancer without lymph-vascular space invasion • Stage 1A/1B/1C grade 1–2 epithelial ovarian cancer • Stage 1A1, 1A2 squamous/adenocarcinoma of the cervix	<5% risk of recurrence	No waiting period after completion of primary treatment	
	Intermediate risk • Stages 1/2 endometrial cancer+risk factors^c^ • Stage 1B squamous/adenocarcinoma of the cervix	5%–15% risk of recurrence	2–3 yr after completion of treatment	
	High risk • Serous, clear cell, or carcinosarcoma of uterus (all stages) • Stage 3 grade 1–3 endometrioid cancer of the uterus • Stage 2/3 epithelial ovarian cancer • Stage 2/3 squamous cell/adenocarcinoma cervical cancer	>30% risk of recurrence	5 yr after completion of treatment	
	Very high risk • Stage 4 endometrial cancer (all grades) • Recurrent or metastatic endometrial cancer • Stage 4 epithelial ovarian cancer (any grade) • Recurrent ovarian cancer • Stage 4 squamous cell/adenocarcinoma of the cervix • Metastatic or recurrent cervical cancer	>80% chance of recurrence	Not a SOT candidate	
RCC	T1a (≤4 cm), N0, M0	95%–98%	No wait time	
	T1b (>4 cm ≤7 cm), N0, M0	91% for FG ½	FG 1–2: no wait time	
		80%–82% for FG ¾	FG 3–4: 1–2 yr	
	T2 (7–10 cm), N0, M0	80%	2 yr	
	T3, N0, M0	43%–80%	Minimum of 2 yr, then reassess	
	T4, N0, M0	28%–55%	Minimum of 2 yr, then reassess	
	Any T, node positive, metastatic disease	0%–32%	Not a candidate (if solitary metastasis resected, tumor board discussion on candidacy	
	Any T with sarcomatoid and/or rhabdoid histologic features	15%–27%	Not a SOT candidate	
	Collecting duct or medullary RCC	<10%	Not a SOT candidate	

Cancer	Risk/Stage/Type	2-yr Local Recurrence from Baseline Trans Urethral Resection of Bladder Tumor	Time Interval to Transplant	
Bladder	Nonmuscle invasive bladder cancer low risk—solitary, ≤3 cm, low grade, Ta tumor, absence of CIS	19%	6 mo	
	Intermediate risk—solitary tumor >3 cm, recurrence within 12 mo with low-grade Ta tumor, multifocal low-grade Ta tumor, low-grade T1 tumor, or high-grade tumor <3 cm	39%	6 mo	
	High risk—any CIS, high-grade Ta tumor >3 cm, high-grade T1 tumor, multifocal high-grade Ta tumor, any recurrent high-grade Ta tumor, CIS, variant histology, LVI, high-grade prostatic urethral involvement, recurrence after BCG intravesical therapy. Although 2-yr recurrence rate is lower than intermediate risk, the progression rate to muscle invasion is higher	38%	2 yr	
	Muscle invasive bladder cancer, postradical cystectomy	25%–37%	2 yr	
	Muscle invasive bladder cancer, postchemoradiation	25%–30% (10 yr)	Not a SOT candidate	

Cancer	Risk/Stage/Type	Survival	Time Interval to Transplant	Additional Considerations
Prostate	Very low risk	<1% risk of mets/death over 15 yr	None	Surveillance is strongly recommended
	• PSA <10 ng/ml			
	• Three or fewer cores of Gleason 6 (grade group 1); no > 50% of individual core			Extenuating circumstances may require treatment
	• T1c–T2a			
	Low risk	Approximately 2%–3% risk of mets/death over 15 years	None	Surveillance is strongly recommended
	• PSA <10 ng/ml			
	• Gleason 6 (not meeting very low-risk criteria)			Extenuating circumstances may require treatment
	• T1c–T2a			
	Low-volume intermediate risk • One of the following criteria: PSA >10 ng/ml, gleason 7 (grade group 2 or 3), T2b	<5% risk of mets/death over 15 yr	If surveillance, no wait time If treatment initiated, and nomogram predicts cancer-specific death over the next 15 yr <10%, no wait time	Surveillance or treatment, depending on patient and cancer characteristics
	High-volume intermediate risk, high-risk, or very high-risk • PSA >20 ng/ml or high-volume Gleason 7 or any Gleason 8–10, T3	20%–70% risk of mets/death over 15 yr	If treatment initiated, and nomogram predicts cancer-specific death over the next 15 yr <10%, no wait time	Treatment
	Metastatic castration-sensitive	Median survival approximately 5–6 years	If stable disease for 2 yr with prolonged estimated life expectancy, may consider transplant	Best systemic therapy±local treatment
	Mestastatic castration-resistant	Median survival 2–3 yr	Not a SOT candidate	Best systemic therapy

Cancer	Risk/Stage/Type		5-yr Disease-Specific Survival	Time Interval to Transplant	Additional Considerations	Appropriate Treatment Pretransplantation
Skin	CSCC	No history of SCC but at risk for development of SCC		No delay necessary	Treatment of field disease	
		Low risk		No delay necessary	Surgical excision with clear margins or Mohs micrographic surgery	
		High-risk SCC (not including PNI)		2 yr	Surgical excision with clear margins or Mohs micrographic surgery	
		High-risk SCC with • PNI or • Two risk factors		2–3 yr	Surgical excision with clear margins or Mohs micrographic surgery ART	
		High risk with local nodal metastatic disease		5 yr	Surgical excision with appropriate lymph node dissection plus ART	
		Distant metastasis		Not eligible for transplantation	Refer for oncology opinion	
	MCC	Local with negative SLNB biopsy		2 yr	Wide local excision ART	
		Local with nodal metastasis		3–5 yr	Wide local excision, lymph node dissection, ART	
		Distant metastasis		Not eligible for transplantation	Refer for oncology opinion	
	MM	*In situ*	99%	No wait time necessary	Follow-up 3 mo post-SOT	Wide local excision
		Stage 1A (T1a)	99%	1 yr		Wide local excision
		Stage 1B (T1b or T2a)	97%	year	If positive SLNB at time of diagnosis, imaging as for stage 2A disease	Wide local excision plus SLNB
		Stage 2A (T2b or T3a)	94%	1 yr	Imaging of the brain, CAP Imaging of the neck for those with head/neck melanoma primary	Wide local excision plus SLNB
		Stage 2B (T3b or T4a)	87%	2–4 yr	Imaging as above	Wide local excision plus SLNB
		Stage 2C (T4b)	82%	2–4 yr	Imaging as above	Wide local excision plus SLNB
		Stage 3A (T1-2a, N1a or 2a)	93%	1–2 yr	Imaging as above oncology referral	Wide excision plus SLNB plus lymph node dissection
		Stage 3B (T0-3a and N1a/b/c, N2a/b)	83%	2–4 yr	Imaging as above oncology referral	Wide excision plus SLNB plus lymph node dissection Adjuvant therapy with CKI
		Stage 3C (T3b-4b and N2b/c-N3b/c)	69%	At least 5 yr	Imaging as above oncology referral (no consensus was possible for this group)	Wide excision plus SLNB plus lymph node dissection Adjuvant therapy with CKI
		Stage 3D (T4b and N3a–3c)	32%	At least 5 yr	Imaging as above oncology referral (no consensus was possible for this group)	Wide excision plus SLNB plus lymph node dissection Adjuvant therapy with CKI
		Stage 4	15%–20%	At least 5 yr	Imaging as above oncology referral (no consensus was possible for this group)	Wide excision plus SLNB plus lymph node dissection Adjuvant therapy with CKI

Adapted from Zwald *et al.* and Al-Adra *et al*.^10,12,13^ ART, adjuvant radiation therapy; BCG, Bacillus Calmette-Guerin; CAP, chest, abdomen, and pelvis; CIS, carcinoma *in situ*; CKI, checkpoint inhibitor; CRC, colorectal cancer; CSCC, cutaneous squamous cell carcinoma; DCIS, ductal carcinoma *in situ*; FG, Fuhrman grade; LVI, lymphovascular invasion; MCC, Merkel cell carcinoma; MM, malignant melanoma; MSI, microsatellite instability; N/A, not applicable; NED, no evidence of disease; PET-CT, positron emission tomography-computed tomography; PNI, perineural invasion; PSA, prostate-specific antigen; RCC, renal cell carcinoma; SBRT, stereotactic body radiation therapy; SCC, squamous cell carcinoma; SLNB, sentinel lymph node biopsy; SOT, solid organ transplantation. Fuhrman grade (grade 1: inconspicuous nucleoli at ×400 magnification and basophilic, grade 2: clearly visible nucleoli at ×400 magnification and eosinophilic, grade 3: clearly visible nucleoli at ×100 magnification, Grade 4: extreme pleomorphism or rhabdoid and/or sarcomatoid morphology).

a After completion of all standard treatments. Endocrine therapy does not need to be completed before transplant, as this is an oral medication that is fairly well tolerated with few serious side effects and often continues for 5–10 years.

b Workup presolid organ transplantation.

c Risk factors: older age, lymph-vascular space invasion, grade 2 or 3 endometrioid, deeply invasive tumor.

## Cancer Screening Post-Kidney Transplant

Because KTRs live longer, they develop *de novo*, recurrent, or donor-derived cancers more frequently. Screening recommendations follow those for the general population. Because there is little evidence available, the decision to screen is based on the judgment of the treating physician.^7^ Although there is some evidence regarding skin and cervical cancers, there is no consensus among international guidelines about screening modality and frequency.^14^

The decision should be tailored, depending on patient comorbidities, life expectancy, cancer risk, and preferences.^7,15^ In Table 2, we present current evidence and a summary of clinical practice guidelines for cancer screening in KTRs.

**Table 2:** Recommendations for cancer screening in kidney transplant recipients

Cancer	Screening	Frequency	Guidelines
Skin and lip	Self-examination	Monthly	AST, KDIGO
		Not specified	CST/CSN, KHA-CARI, UKKA
	Examination by primary care physician or dermatologist	Annually^a^	AST, CST/CSN, KDIGO, KHA-CARI, UKKA
Cervical	Pelvic examination^b^	Every 3 yr	KDIGO, UKKA
		Annually	AST, CST/CSN, EBPG
		Not specified	KHA-CARI
	Pap smear^b^	Every 3 yr	KDIGO, UKKA
		Annually	AST, CST/CSN, EBPG
		Not specified	KHA-CARI
Colorectal	FOBT^c^	Follow general population guidelines (annually)	AST, CST/CSN, KDIGO, UKKA, EBPG, KHA-CARI
	Sigmoidoscopy^c^	Follow general population guidelines (every 5 yr)	AST, CST/CSN, KDIGO
	Colonoscopy^c^	Follow general population guidelines (every 10 yr)	AST, CST/CSN, EBPG, KDIGO, UKKA
RCC	Not recommended		AST, CST/CSN, KDIGO, UKKA, KHA-CARI
	Ultrasound^d^	Annually	EAU
		Not specified	EBPG
Prostate	Digital rectal examination^e^	Annually	AST, EBPG
		Follow general population guidelines^f^	CST/CSN, KDIGO, KHA-CARI, UKKA
	PSA^e^	Annually	AST, EBPG
		Follow general population guidelines^f^	CST/CSN, KDIGO, KHA-CARI, UKKA
Breast	Mammography^g^	1–2 yr	AST
		Follow general population guidelines	CST/CSN, KDIGO, RA, EBPG, KHA-CARI
Post-transplantation lymphoproliferative disorders	Physical examination	Every 3 mo in the first year, then annually	AST
	EBV viremia^h^	Once in the first week after transplantation, monthly for the first 3–6 mo and then every 3 mo until the end of the first post-transplant year	KDIGO, UKKA
Hepatocellular carcinoma	*α*-fetoprotein^i^	Annually	CST/CSN, KDIGO, UKKA
		Every 6–12 mo	AST
	Abdominal ultrasound^i^	Annually	CST/CSN, KDIGO, UKKA
		Every 6–12 mo	AST
Lung		Not recommended	AST, CST/CSN, EBPG, KDIGO, KHA-CARI, UKKA

AST, American Society of Transplantation; CST/CSN, Canadian Society of Transplantation and the Canadian Society of Nephrology; EAU, European Association of Urologists; EBPG, European Best Practice Guidelines; EBV, Epstein-Barr virus; FOBT, fecal occult blood test; KDIGO, Kidney Disease Improving Global Outcomes; KHA-CARI, Kidney Health Australia–Caring for Australasians with Renal Impairment; Pap, Papanicolaou; PSA, prostate-specific antigen; RCC, renal cell carcinoma; UKKA, United Kingdom Kidney Association.

a The United Kingdom Kidney Association recommends at least biannual review by a specialist up to 5 years post-transplant and annual review from 5 years onward.

b Screening age is variable among guidelines. Kidney Disease Improving Global Outcomes recommends initiation of screening for women within 3 years of onset of sexual activity or age 21 until age 65 years (if previous examinations have no abnormality); American Society of Transplantation recommends for women older than 18 years. The United Kingdom Kidney Association recommends screening every 3 years between age 25 and 49 years and every 5 years between age 50 and 64 years.

c American Society of Transplantation and Kidney Disease Improving Global Outcomes recommend annual fecal occult blood test in adults older than 45 years; the United Kingdom Kidney Association recommends every 2 years in adults aged 60–74 years; European Best Practice Guidelines advocates using fecal occult blood test with no specific frequency in adults older than 50 years. High-risk patients might benefit from earlier and more frequent screening.

d European Association of Urologists recommends annual screening and the European Best Practice Guidelines recommends native renal cell carcinoma screening using ultrasonography, with variable frequency between 1 and 3 years based on expected growth rate of tumors.

e Screening in adults 50 years and older with at least 10-year expected life span.

f In Australia and the United Kingdom, there is no national screening program for prostate cancer. The US Preventive Services Task Force recommends screening between age 50 and 68 and the Canadian Urological Association advocates screening in age 50 for most men and age 45 in men at increased risk of prostate cancer.^16,17^

g American Society of Transplantation recommends mammography screening at age 50–60; the US Preventive Services Task Force, Australian and Canadian national guidelines recommend mammography every 2 years in women aged 50–74 years, the National Health Service (United Kingdom) recommends screening every 3 years for women aged 50–70 years.^18–21^

h Only for high-risk patients (donor seropositive/recipient seronegative).

i Only for patients with liver disease.

### Skin and Lip

KDIGO and the United Kingdom Kidney Association (UKKA) recommend KTRs to be informed about their high-risk skin cancer status, especially patients with fair skin tones, who reside in areas with high ultraviolet exposures, have occupations requiring sun exposure, and have a history of skin cancer.^22,23^ All patients should minimize life-long sun exposure and use appropriate ultraviolet sunscreen and hats.^22,24,25^ Self-examination and annual clinical skin examination are recommended, whether by a primary care physician or a dermatologist.^22,25–28^ A tailored surveillance program is recommended on the basis of risk stratification.^15^ A Delphi survey advised screening within 2 (high-risk patients) to 5 years (low-risk patients) of transplant, without specifying ongoing screening intervals.^29^

### Cervical

KDIGO and the UKKA recommend Papanicolaou cytology with pelvic examination every 3 years in patients between ages 21 and 65 years, according to general population recommendations.^22,23^ However, the optimal frequency for KTRs remains undetermined. Canadian Society of Transplantation and the Canadian Society of Nephrology (CST/CSN), the Kidney Health Australia–Caring for Australasians with Renal Impairment (KHA-CARI), AST, and European Best Practice Guidelines (EBPG) guidelines recommend more frequent screening with annual pelvic examination and Pap smear.^24,25,27,28^ In addition, a visual inspection of the anal and vulvar area should be included.^30^ The AST Infectious Diseases Community of Practice suggested that transplant recipients should be screened with the same periodicity as patients with HIV because of their immunodeficient status.^31^

Some guidelines advocate the use of human papillomavirus (HPV) DNA testing alongside cytological screening for women aged older than 30 years in the general population.^7^ However, there is limited evidence supporting its application in KTRs. Some physicians use HPV testing at 1 year, and, if positive, screening continues every 6 months.^6,31^ Studies demonstrating the benefits of HPV vaccines in transplant cohorts remain limited.^32^ According to the Pediatric Transplantation Association, vaccination in the SOT should differ from the general population.^33^ SOT recipients should receive three doses of HPV vaccine, regardless of age. Vaccination can be considered in adult SOT recipients between age 26 and 45 years.^33^ Vaccination is more effective before transplantation, and if administered post-transplant, it should be administered 3–6 months post-transplant to enhance immune response.^34^

### Colorectal

AST, CST/CSN, KDIGO, and UKKA recommend screening colorectal cancer (CRC) in KTRs similarly to the general population, and EBPG advocates only fecal occult blood tests without specification for frequency.^14,22,24,25,28^ Guidelines for the general population at average risk for CRC recommend initiating screening at age 50 years. However, the increasing incidence of CRC in the younger population has prompted the American Cancer Society to lower the starting age to 45 years. Screening involves annual high-sensitivity guaiac fecal occult blood test or fecal immunochemical test, stool DNA-fecal immunochemical test testing every 1–3 years, a flexible sigmoidoscopy every 5 years, or colonoscopy every 10 years.^6,35^ It is reasonable to consider adjusting the intervals of colonoscopy between 5 and 10 years for post-transplant patients, tailored to individual factors for CRC.^36^ High-risk patients or patients exposed to high-intensity immunosuppression may benefit from screening at earlier age or with shorter intervals.^36^ The choice of CRC screening modalities are most frequently at the discretion of patient-clinician discussions, and no consensus guidelines are available from transplant organizations.

### RCC

KTRs are at elevated risk of RCC in native kidneys. Current screening recommendations are not consensual. AST and KDIGO guidelines do not recommend screening for RCC.^22,25,37^ The European Association of Urologists recommends annual evaluation, and the EBPG recommends native RCC regular screening using ultrasonography, with no specification about frequency.^24,37,38^

RCC screening should be individualized and focus on high-risk subgroups, such as those with a family history of renal cancer, acquired cystic disease, or familial syndrome.^24,37–39^

The optimal screening modality for RCC is yet to be determined. Despite being less expensive and widely available, ultrasound is operator dependent, may not detect early lesions, and its quality is limited by patient-related factors such as obesity. Contrast-enhanced computed tomography (CT) is the gold standard to detect renal mass and might be particularly effective in acquired kidney disease. However, it presents higher cost and risk of radiation exposure and contrast nephropathy.^40^

### Prostate

CST/CSN, KDIGO, KHA-CARI, and UKKA follow general population guidelines.^14,22,27,28^ AST and EBPG recommend annual digital rectal examination and prostate-specific antigen testing for male patients older than 50 years and older with at least 10-year expected life span.^24,25^ The AST guidelines add that screening at an earlier age might be reasonable for men with individual risk factors such as a family history of prostate cancer.^25^

### Breast

CST/CSN, KDIGO, KHA-CARI, EBPG, and UKKA recommend KTRs to follow general population guidelines.^14,22,28^ AST recommends average-risk women to begin screening at age 50 years with mammography every 1–2 years. Screening between 40 and 49 years should be based on individual risk and coexisting comorbidities.^25,41^

### PTLDs

AST recommends history and physical examination for constitutional symptoms and lymphadenopathy/organomegaly every 3 months in the first post-transplant year and annually thereafter.^25^ Most transplant programs test for viremia in Epstein-Barr virus (EBV) mismatched KTRs (donor seropositive/recipient seronegative). If rising EBV load is identified in seronegative KTRs, immunosuppression should be reduced to prevent EBV-related diseases and PTLD.^22,25,30,42^ Evidence is currently insufficient to support the use of preemptive rituximab or antiviral agents.^43^

EBV viremia should also be evaluated after acute rejection treatment.^22^

### Hepatocellular Carcinoma

AST, CST/CSN, EBPG, and KDIGO recommend that high-risk individuals, including those with cirrhosis, liver disease, or chronic viral infection (such as hepatitis B or hepatitis C viruses), undergo screening using *α*-fetoprotein and ultrasound every 6–12 months.^22,23,25,28^ CT improves the ability to characterize small hepatic lesions and diagnose early stage hepatocellular cancer, although to our knowledge, there are no studies evaluating the test performance of CT or magnetic resonance imaging for screening KTRs.^25^

### Lung

Previous trials conducted in the general population, using *x* ray alone or in combination with sputum cytology, failed to demonstrate any mortality benefits in the screened population.^44^ Therefore, AST, CST/CSN, KDIGO, KHA-CARI, and UKKA do not currently recommend lung cancer screening in KTRs.^22,25,27,28^ However, more recent evidence suggests that high-risk general population individuals benefit from annual screening.^45^ The American Cancer Society and the US Preventive Services Task Force advocate annual screening with low-dose CT in individuals aged 50–80 years with a ≥20 pack-year history of smoking or current smokers.^46,47^ The effectiveness of low-dose CT in high-risk KTRs remains unclear.^25^

## Cancer Treatment

### General Approach of Cancer Treatment in KTRs

In KTRs diagnosed with cancer, treatment options encompass conventional multimodal therapies, including radiotherapy and chemotherapy, combined with alterations in immunosuppressants. Cumulative immunosuppression is the primary factor to determine the risk of cancer in KTRs, and reduction in immunosuppression is considered as the first step to promote antitumor immune response. Nevertheless, there is no consensus regarding the optimal approach for reducing immunosuppressants during cancer treatment in KTRs. The permissible degree and effectiveness of immunosuppressant dose reduction vary depending on the type and stage of the underlying cancer. In addition, this approach needs to be carefully weighed up against the risk of graft rejection. Collaboration among transplant nephrologists and oncologists is imperative to ensure optimal patient care.

Alterations in immunosuppressants could affect the risk of cancer in KTRs. Calcineurin inhibitors (CNIs) can promote cancer progression through several mechanisms, including the production of TGF-*β*,^48^ vascular endothelial growth factor,^49^ and activation of the rat sarcoma-rapidly accelerated fibrosarcoma pathway.^50^ Several studies have indicated that switching to mammalian target of rapamycin (mTOR) inhibitors reduces the risk of cancer in KTRs, as discussed in the following section. In KTRs, the use of IL-2 receptor antagonists (*e.g*., basiliximab)^51^ is associated with a lower risk of cancer, compared with depleting induction therapies such as antithymoglobulin and muromonab-CD3.^52^ Azathioprine use is associated with the increased risk of cancer, especially cutaneous squamous cell carcinoma (CSCC) in KTRs.^53^ On the other hand, mycophenolate mofetil may be associated with a lower risk of cancer in KTRs compared with azathioprine.^54^ Belatacept, a costimulatory blockade agent, has been routinely used as immunosuppression post-transplant. Initial trials indicated clearly elevated risk of PTLD in patients seronegative for EBV, which led to a black box warning by US Food and Drug Administration.^55,56^ However, studies suggested conflicting data on the effect of belatacept on the incidence of post-transplant malignancy. Cochrane review,^57^ based on the pooled data from Belatacept Evaluation of Nephroprotection and Efficacy as First-line Immunosuppression Trial and BENEFIT-EXTended criteria donors studies, indicated the effect of belatacept on post-transplant malignancy was inconclusive, except for PTLD. For CSCC, an initial single-center study^58^ suggested a lower incidence of CSCC in patients treated with belatacept compared with CNI, but recent studies showed^59,60^ an equivocal incidence of CSCC for patients on belatacept compared with others.

The choice and dosing of chemotherapeutic agents should be carefully determined according to allograft function and potential interactions between drugs. Several chemotherapeutic agents have been reported to increase allograft rejection rate. High-dose IL-2 therapy, which has been used to treat metastatic RCC, could increase rejection rate in KTRs through robust effector T-cell activation.^61^ Immunomodulatory drugs, such as lenalidomide and pomalidomide used against multiple myeloma, are associated with increased rejection rate because of direct activation of costimulatory signaling (CD28) of T cells.^62,63^ Anti-vascular endothelial growth factor therapy can induce thrombotic microangiopathy in the allograft and in native kidneys.^64^ The efficacy and tolerability of newer anticancer treatments, exemplified by immune checkpoint inhibitors (ICIs) and cell therapies (notably chimeric antigen receptor T [CAR-T]-cell therapy), have also been investigated in KTRs, and we will discuss these novel treatments in the subsequent sections by reviewing updates in two clinical contexts that highlight cancer management in KTRs: skin cancer and PTLD.

### Skin Cancer in KTRs

Compared with the general population, KTRs are at higher risk of developing skin cancer, especially CSCC and basal cell carcinoma (BCC).^65^ The standardized incidence ratio (SIR) of skin cancer in KTRs is approximately 65–250 (CSCC), 10–16 (BCC), 0–8 (melanoma), and 84–500 (Kaposi sarcoma) compared with the age-matched and sex-matched general population.^42^ CSCC is the most common type of skin cancer in KTRs followed by BCCs, while this ratio is reversed in the general population. The mortality in KTRs by CSCC and BCC is over 20 times higher than that in the general population.^66^ While melanoma is less common than CSCC and BCC, the risk of melanoma remains higher in KTRs than in the general population.^67^ Recurrent or multiple site skin cancer is common in KTRs, with a cumulative incidence of secondary skin cancer reaching 59% in 3 years after the first skin cancer.^68^

Choice of immunosuppressants affects the incidence of skin cancer in KTRs. Regimens including mTOR inhibitors such as sirolimus and everolimus rather than CNIs reduce the risk of NMSC and delay the onset in KTRs.^69^ In a meta-analysis including 39,039 KTRs, sirolimus use was associated with the reduction in the incidence of NMSC with incidence rate ratio of 0.49 (95% confidence interval [CI], 0.32 to 0.76).^70^ This was partly attributable to the withdrawal of cyclosporine because the protective effect of sirolimus was most notable in studies comparing sirolimus against cyclosporine (incidence rate ratio, 0.19; 95% CI, 0.04 to 0.84). Indeed, in a mouse model of ultraviolet-induced skin cancer, larger tumors developed in mice treated with cyclosporine or tacrolimus than in those treated with sirolimus.^71^ mTOR inhibitors are effective in secondary prevention of skin cancer in KTRs because conversion from CNIs to sirolimus decreased the risk of secondary skin cancer in KTRs with previous CSCC.^72^ Another study, however, showed a higher mortality rate in patients receiving mTOR inhibitors, which was attributable to increased cardiovascular and infection-related deaths (hazard ratio, 1.43; 95% CI, 1.21 to 1.71).^73^ Therefore, switching to mTOR inhibitors should be carefully determined considering risks of cardiovascular events and benefits of cancer prevention. Although evidence is limited, mycophenolate mofetil is also reported to be associated with a lower incidence of skin cancer in SOT recipients compared with azathioprine.^74^ Another option to prevent skin cancer in KTRs is the use of chemoprotective agents such as acitretin and nicotinamide, but evidence is scarce.

ICIs have demonstrated favorable outcomes as a novel treatment for various types of malignancies. ICI might improve outcomes in KTRs with skin cancer whereas it could increase the risk of graft rejection. In a multicenter retrospective study of KTRs receiving ICI, 42% of the patients developed acute rejection, a rate significantly higher than that of the non–ICI-treated cohort (5.4%).^75^ Despite high rejection rate, patients with CSCC treated with ICI exhibited a significantly longer overall survival (median overall survival of 19.8 versus 10.6 months), suggesting that ICI might be useful in specific subpopulations of KTRs. The optimal immunosuppressant regimen to prevent rejection under ICI use has not yet been confirmed; however, a recent study has shown a promising regimen (Table 3).^76–79^ In a phase 1 clinical trial testing cemiplimab in KTRs with advanced CSCC, a combination of mTOR inhibitors and corticosteroids completely avoided graft rejection or loss, with durable antitumor responses.^78^ Future study is needed to solidify the data on favorable approaches of immunosuppression under ICI treatment.

**Table 3:** Summary of clinical trials on the optimal immunosuppressant regimen to prevent rejection using immune checkpoint inhibitors

Cancer Type	Any Cancers (Incurable, Metastatic Tumors)	Skin Cancers (Melanoma, CSCC, BCC, MCC)	CSCC
Transplant	Kidney	Kidney	Kidney
ICI	Nivolumab	Nivolumab±ipilimumab	Cemiplimab
Immunosuppression	Keep the same dose	Tac (2–5 ng/ml)+Pred 5 mg/d	mTOR inhibitors+dynamic Pred
No. of patients	17	8	12
Rejection	2 (11.7%)	2 (25%)	0 (0%)
Overall response rate	53%	33%	45%
Registry	ANZCTR CA209-9931SR	NCT03816332	NCT04339062
Country	Australia	US	US
Reference	*Lancet Oncol* (2022)^76^	*J Clin Oncol* (2024)^77^	*J Clin Oncol* (2024)^78^

Adapted from Van Meerhaeghe *et al*.^79^ Immune checkpoint inhibitor use improves outcomes of kidney transplant recipients with cancer; however, it may increase the risk of graft rejection. Recent clinical trials have shown that optimizing immunosuppressant therapy can mitigate the risk of rejection during immune checkpoint inhibitor use. Combination of mammalian target of rapamycin inhibitors with pulsed-dose corticosteroids prevented rejection in kidney transplant recipients with advanced cutaneous squamous cell carcinoma that were treated with cemiplimab. ANZCTR, Australian New Zealand Clinical Trials Registry; BCC, basal cell carcinoma; BRAF, v-Raf murine sarcoma viral oncogene homolog B1; CSCC, cutaneous squamous cell carcinoma; ICI, immune checkpoint inhibitors; KTR, kidney transplant recipients; MCC, Merkel cell carcinoma; mTOR, mammalian target of rapamycin; Pred, prednisolone; Tac, tacrolimus.

### Post-Transplant Lymphoproliferative Disease in KTRs

PTLD is one of the life-threatening complications after transplantation characterized by abnormal lymphoproliferation. Sprangers *et al.* provided a comprehensive summary of PTLD in KTRs in their review article.^80^ The lifetime risk of lymphoproliferative disease is 30-fold higher in childhood recipients (SIR, 29.5; 95% CI, 21.9 to 38.8) and eight-fold higher in adult recipients (SIR, 8.4; 95% CI, 7.7 to 9.2) than the age-matched general population.^81^ In a retrospective cohort study including pediatric recipients of SOT, the risk of cancer after transplantation, among which PTLD was the most common, was highest in the first year post-transplant (adjusted hazard ratio, 176; 95% CI, 117 to 264) and remained elevated beyond 10 years (adjusted hazard ratio, 10.8; 95% CI, 6.3 to 18.6).^82^ PTLD has bimodal presentation peaks—early presentation within 1 year post-transplant (commonly EBV positive) and late presentation later than 10 years post-presentation (commonly EBV negative; 38%–48%). EBV-associated transformation of B cells plays key roles on the pathogenesis of PTLD, by expression of EBV-related proteins, including membrane proteins (latent membrane protein 1/2A) and nuclear EBV proteins (epstein-barr virus nuclear antigen-2 and EBNA-leader protein), which induce abnormal B-cell growth and proliferation. CNIs and depleting induction therapy (*e.g*., antithymoglobulin) are associated with increased risk of PTLD.^83,84^

Clinical presentation of PTLD is highly variable, most commonly affecting lymph nodes, gastrointestinal tract, and central nervous system and allograft.^80,85^ According to the 2017 World Health Organization criteria, PTLD is categorized into four subtypes: early lesions (plasmacytic hyperplasia and infectious mononucleosis-like lesions), polymorphic PTLD, monomorphic PTLD, and classical Hodgkin lymphoma–like PTLD.^86^ In a recently updated version (the fifth edition of the World Health Organization classification), PTLD is classified within a more comprehensive category of immunodeficiency-associated lymphoproliferative disorders, which arise in various contexts of immune dysfunction, rather than being solely based on morphological classification.^80,85,87^ The mainstay of treatment for PTLD is reduction in immunosuppression,^88^ but a combination with systemic chemotherapy/targeted therapy, radiation or surgery is sometimes necessary. In patients with CD20-positive PTLD, rituximab has become a standard treatment option. In KTRs, rituximab combined with cyclophosphamide, doxorubicin, vincristine, prednisolone chemotherapy reduced the risk of renal graft impairment.^89^ The effectiveness of single-agent rituximab has also been reported.^90^

Recently, CAR-T therapy, which has shown substantial efficacy in patients with relapsed/refractory diffuse large B-cell lymphoma,^91,92^ has been used in patients with refractory PTLD. In the earlier case series, however, the outcome was unfavorable: Most of the patients failed to show response to CAR-T therapy and developed complications, including cytokine release syndrome and immune effector cell-associated neurotoxicity syndrome.^93,94^ Conversely, more recent studies have reported promising results, showing good response to CAR-T therapy while maintaining low rejection rate.^95,96^ It seems that an induction therapy with cyclophosphamide before CAR-T therapy following minimum dose of immunosuppression (*e.g*., prednisone 5–10 mg/d) reduces the risk of rejection while sustaining efficacy of CAR-T therapy. In a multicenter, retrospective analysis of patients with relapsed/refractory PTLD treated by CAR-T therapy, the 2-year progression-free survival, and overall survival rates were 35% and 58%, respectively, which is similar to the previously reported result of CAR-T therapy on patients with diffuse large B-cell lymphoma.^97^ Future studies are necessary to better understand the effectiveness and tolerability of CAR-T therapy in patients with PTLD.

## Future Directions

In the era of precision medicine, pretransplant and post-transplant cancer screening using noninvasive and sensitive molecular diagnostics will become increasingly important. For example, circulating tumor DNA testings have been integrated in clinics to detect molecular residual disease of solid tumors, such as lung cancer,^98,99^ and CRC.^100–102^ Similarly, minimal/measurable residual disease testing is a next-generation sequence-based diagnostic test to detect a very small number of cancer cells in hematological malignancy, such as multiple myeloma,^103–105^ leukemia,^106^ and lymphoma.^107^ These molecular diagnostics can be useful to ensure a stringent cancer-free condition pretransplant and can be used to monitor post-transplant cancer recurrence. Furthermore, the long-term outcomes of premalignant conditions such as pancreatic neuroendocrine tumor, gastrointestinal stromal tumor, smoldering myeloma/monoclonal gammopathy of undetermined significance and clonal hematopoiesis of indeterminate potential, especially under long-term chronic immunosuppression, will need to be further studied in the future to tailor immunosuppression regimens on the basis of individuals' cancer risks.^108^

## Conclusion

Pretransplant malignancy is increasingly common in kidney transplant candidates and can affect post-transplant outcomes. Although cancer management after transplantation is challenging and relies on a fine balance of immunosuppression reduction, novel therapies and improved outcomes give us some hope. Because KTRs are generally excluded from cancer-related clinical trials, prospective data from patients with malignancies before and after transplantation, as historically done in the Israel Penn International Transplant Tumor Registry, should ideally be collected, shared, and analyzed to allow the development of standardized screening guideline.^109^ We call for further research to evaluate how cancer screening, risk factor prevention, modalities of immunosuppression reduction, and novel cancer therapies affect outcomes.

## References

[1] Ietto G Gritti M Pettinato G Carcano G Gasperina DD. Tumors after kidney transplantation: a population study. World J Surg Oncol. 2023;21(1):18. doi:10.1186/s12957-023-02892-3 36691019 PMC9869548

[2] Vajdic CM McDonald SP McCredie MRE, . Cancer incidence before and after kidney transplantation. JAMA. 2006;296(23):2823–2831. doi:10.1001/jama.296.23.2823 17179459

[3] Villeneuve PJ Schaubel DE Fenton SS Shepherd FA Jiang Y Mao Y. Cancer incidence among Canadian kidney transplant recipients. Am J Transplant. 2007;7(4):941–948. doi:10.1111/j.1600-6143.2007.01736.x 17331115

[4] Au E Wong G Chapman JR. Cancer in kidney transplant recipients. Nat Rev Nephrol. 2018;14(8):508–520. doi:10.1038/s41581-018-0022-6 29802400

[5] Miao Y Everly JJ Gross TG, . De novo cancers arising in organ transplant recipients are associated with adverse outcomes compared with the general population. Transplantation. 2009;87(9):1347–1359. doi:10.1097/TP.0b013e3181a238f6 19424035

[6] Dharia A Boulet J Sridhar VS Kitchlu A. Cancer screening in solid organ transplant recipients: a focus on screening liver, lung, and kidney recipients for cancers related to the transplanted organ. Transplantation. 2022;106(1):e64–e65. doi:10.1097/TP.0000000000003773 33795594

[7] Wong G Chapman JR Craig JC. Cancer screening in renal transplant recipients: what is the evidence? Clin J Am Soc Nephrol. 2008;3(suppl 2):S87–S100. doi:10.2215/CJN.03320807 18309007 PMC3152279

[8] Wong G Hayward JS McArthur E, . Patterns and predictors of screening for breast and cervical cancer in women with CKD. Clin J Am Soc Nephrol. 2017;12(1):95–104. doi:10.2215/CJN.05990616 28034851 PMC5220661

[9] Chadban SJ Ahn C Axelrod DA, . KDIGO clinical practice guideline on the evaluation and management of candidates for kidney transplantation. Transplantation. 2020;104(4S1 suppl 1):S11–S103. doi:10.1097/TP.0000000000003136 32301874

[10] Zwald F Leitenberger J Zeitouni N, . Recommendations for solid organ transplantation for transplant candidates with a pretransplant diagnosis of cutaneous squamous cell carcinoma, Merkel cell carcinoma and melanoma: a consensus opinion from the International Transplant Skin Cancer Collaborative (ITSCC). Am J Transplant. 2016;16(2):407–413. doi:10.1111/ajt.13593 26820755

[11] Serkies K Dębska-Ślizień A Kowalczyk A Lizakowski S Małyszko J. Malignancies in adult kidney transplant candidates and recipients: current status. Nephrol Dial Transplant. 2023;38(7):1591–1602. doi:10.1093/ndt/gfac239 35998321

[12] Al-Adra DP Hammel L Roberts J, . Pretransplant solid organ malignancy and organ transplant candidacy: a consensus expert opinion statement. Am J Transplant. 2021;21(2):460–474. doi:10.1111/ajt.16318 32969590 PMC8576374

[13] Al-Adra DP Hammel L Roberts J, . Preexisting melanoma and hematological malignancies, prognosis, and timing to solid organ transplantation: a consensus expert opinion statement. Am J Transplant. 2021;21(2):475–483. doi:10.1111/ajt.16324 32976703 PMC8555431

[14] Manickavasagar R Thuraisingham R. Post renal-transplant malignancy surveillance. Clin Med. 2020;20(2):142–145. doi:10.7861/clinmed.2019-0423 PMC7081823 32188647

[15] Acuna SA Huang JW Scott AL, . Cancer screening recommendations for solid organ transplant recipients: a systematic review of clinical practice guidelines. Am J Transplant. 2017;17(1):103–114. doi:10.1111/ajt.13978 27575845

[16] Grossman DC Curry SJ Owens DK, .; US Preventive Services Task Force. Screening for prostate cancer: US preventive Services Task Force recommendation statement. JAMA. 2018;319(18):1901–1913. doi:10.1001/jama.2018.3710 29801017

[17] Mason RJ Marzouk K Finelli A, . Update - 2022 Canadian Urological Association recommendations on prostate cancer screening and early diagnosis Endorsement of the 2021 Cancer Care Ontario guidelines on prostate multiparametric magnetic resonance imaging. Can Urol Assoc J. 2022;16(4):E184–E196. doi:10.5489/cuaj.7851 35358414 PMC9054332

[18] Siu AL.; US Preventive Services Task Force. Screening for breast cancer: U.S. Preventive Services Task Force recommendation statement. Ann Intern Med. 2016;164(4):279–296. doi:10.7326/M15-2886 26757170

[19] Klarenbach S Sims-Jones N Lewin G, . Recommendations on screening for breast cancer in women aged 40-74 years who are not at increased risk for breast cancer. CMAJ. 2018;190(49):E1441–E1451. doi:10.1503/cmaj.180463 30530611 PMC6279444

[20] Royal Australian College of General Practitioners. National Preventive and Community Medicine Committee. Guidelines for preventive activities in general practice, 9th Ed, 2018. https://www.racgp.org.au/FSDEDEV/media/documents/Clinical%20Resources/Guidelines/Red%20Book/Guidelines-for-preventive-activities-in-general-practice.pdf

[21] Williams J Garvican L Tosteson ANA Goodman DC Onega T. Breast cancer screening in England and the United States: a comparison of provision and utilisation. Int J Public Health. 2015;60(8):881–890. doi:10.1007/s00038-015-0740-5 26446081 PMC6525304

[22] Kidney Disease Improving Global Outcomes (KDIGO) Transplant Work Group. KDIGO clinical practice guideline for the care of kidney transplant recipients. Am J Transplant. 2009;9(suppl 3):S1–S155. doi:10.1111/j.1600-6143.2009.02834.x 19845597

[23] Baker RJ Mark PB Patel RK Stevens KK Palmer N. Renal association clinical practice guideline in post-operative care in the kidney transplant recipient. BMC Nephrol. 2017;18(1):174. doi:10.1186/s12882-017-0553-2 28571571 PMC5455080

[24] EBPG Expert Group on Renal Transplantation. European best practice guidelines for renal transplantation. Section IV: long-term management of the transplant recipient. IV.6.3. Cancer risk after renal transplantation. Solid organ cancers: prevention and treatment. Nephrol Dial Transplant. 2002;17(suppl 4):34–36. PMID: 12091641 12091641

[25] Kasiske BL Vazquez MA Harmon WE, . Recommendations for the outpatient surveillance of renal transplant recipients. J Am Soc Nephrol. 2000;11(suppl 15):S1–S86. doi:10.1681/ASN.v11suppl_1s1 11044969

[26] Bia M Adey DB Bloom RD Chan L Kulkarni S Tomlanovich S. KDOQI US commentary on the 2009 KDIGO clinical practice guideline for the care of kidney transplant recipients. Am J Kidney Dis. 2010;56(2):189–218. doi:10.1053/j.ajkd.2010.04.010 20598411

[27] Chadban SJ Barraclough KA Campbell SB, . KHA-CARI guideline: KHA-CARI adaptation of the KDIGO clinical practice guideline for the care of kidney transplant recipients. Nephrology. 2012;17(3):204–214. doi:10.1111/j.1440-1797.2011.01559.x 22212251

[28] Knoll GA Blydt-Hansen TD Campbell P, . Canadian Society of Transplantation and Canadian Society of Nephrology commentary on the 2009 KDIGO clinical practice guideline for the care of kidney transplant recipients. Am J Kidney Dis. 2010;56(2):219–246. doi:10.1053/j.ajkd.2010.05.004 20659623

[29] Crow LD Jambusaria-Pahlajani A Chung CL, . Initial skin cancer screening for solid organ transplant recipients in the United States: delphi method development of expert consensus guidelines. Transpl Int. 2019;32(12):1268–1276. doi:10.1111/tri.13520 31502728

[30] Asch WS Bia MJ. Oncologic issues and kidney transplantation: a review of frequency, mortality, and screening. Adv Chronic Kidney Dis. 2014;21(1):106–113. doi:10.1053/j.ackd.2013.07.003 24359993

[31] Chin-Hong PV Kwak EJ.; AST Infectious Diseases Community of Practice. Human papillomavirus in solid organ transplantation. Am J Transplant. 2013;13(suppl 4):189–200. doi:10.1111/ajt.12142 23465011

[32] Silverberg MJ Leyden WA Lam JO, . Effectiveness of “catch-up” human papillomavirus vaccination to prevent cervical neoplasia in immunosuppressed and non-immunosuppressed women. Vaccine. 2020;38(29):4520–4523. doi:10.1016/j.vaccine.2020.05.004 32446836 PMC7272293

[33] Nailescu C Ermel AC Shew ML. Human papillomavirus-related cancer risk for solid organ transplant recipients during adult life and early prevention strategies during childhood and adolescence. Pediatr Transplant. 2022;26(7):e14341. doi:10.1111/petr.14341 35808949

[34] Chin-Hong PV Reid GE.; AST Infectious Diseases Community of Practice. Human papillomavirus infection in solid organ transplant recipients: guidelines from the American Society of Transplantation infectious diseases community of practice. Clin Transplant. 2019;33(9):e13590. doi:10.1111/ctr.13590 31077438

[35] Lin JS Perdue LA Henrikson NB Bean SI Blasi PR. Screening for colorectal cancer: updated evidence report and systematic review for the US preventive Services Task Force. JAMA. 2021;325(19):1978–1998. doi:10.1001/jama.2021.4417 34003220 PMC13509038

[36] Prenner S Levitsky J. Comprehensive review on colorectal cancer and transplant. Am J Transplant. 2017;17(11):2761–2774. doi:10.1111/ajt.14340 28471512

[37] Wong G Howard K Webster AC Chapman JR Craig JC. Screening for renal cancer in recipients of kidney transplants. Nephrol Dial Transplant. 2011;26(5):1729–1739. doi:10.1093/ndt/gfq627 20961889

[38] Rodríguez Faba O Boissier R Budde K, . European association of urology guidelines on renal transplantation: update 2018. Eur Urol Focus. 2018;4(2):208–215. doi:10.1016/j.euf.2018.07.014 30033070

[39] Yohannan B Sridhar A Kaur H DeGolovine A Maithel N. Screening for renal cell carcinoma in renal transplant recipients: a single-centre retrospective study. BMJ Open. 2023;13(9):e071658. doi:10.1136/bmjopen-2023-071658 PMC10503370 37699639

[40] Diana P Klatte T Amparore D, . Screening programs for renal cell carcinoma: a systematic review by the EAU young academic urologists renal cancer working group. World J Urol. 2023;41(4):929–940. doi:10.1007/s00345-022-03993-6 35362747 PMC10160199

[41] Wong G Au E Badve SV Lim WH. Breast cancer and transplantation. Am J Transplant. 2017;17(9):2243–2253. doi:10.1111/ajt.14368 28544474

[42] Al-Adra D Al-Qaoud T Fowler K Wong G. De novo malignancies after kidney transplantation. Clin J Am Soc Nephrol. 2022;17(3):434–443. doi:10.2215/CJN.14570920 33782034 PMC8975024

[43] Allen UD Preiksaitis JK.; AST Infectious Diseases Community of Practice. Epstein-Barr virus and posttransplant lymphoproliferative disorder in solid organ transplantation. Am J Transplant. 2013;13(suppl 4):107–120. doi:10.1111/ajt.12104 23465004

[44] Manser RL Irving LB Stone C Byrnes G Abramson M Campbell D. Screening for lung cancer. Cochrane Database Syst Rev. 2013;2013(6):CD001991. doi: 10.1002/14651858.CD001991.pub3 14973979

[45] de Koning HJ van der Aalst CM de Jong PA, . Reduced lung-cancer mortality with volume CT screening in a randomized trial. N Engl J Med. 2020;382(6):503–513. doi:10.1056/NEJMoa1911793 31995683

[46] Wolf AMD Oeffinger KC Shih TYC, . Screening for lung cancer: 2023 guideline update from the American Cancer Society. CA Cancer J Clin. 2024;74(1):50–81. doi:10.3322/caac.21811 37909877

[47] Sridhar A Yohannan B Kaur H. Characteristics of lung cancer in renal transplant recipients. J Clin Oncol. 2023;41(16 suppl):e20592. doi:10.1200/jco.2023.41.16_suppl.e20592

[48] Hojo M Morimoto T Maluccio M, . Cyclosporine induces cancer progression by a cell-autonomous mechanism. Nature. 1999;397(6719):530–534. doi:10.1038/17401 10028970

[49] Basu A Contreras AG Datta D, . Overexpression of vascular endothelial growth factor and the development of post-transplantation cancer. Cancer Res. 2008;68(14):5689–5698. doi:10.1158/0008-5472.CAN-07-6603 18632621 PMC2630878

[50] Datta D Contreras AG Basu A, . Calcineurin inhibitors activate the proto-oncogene Ras and promote protumorigenic signals in renal cancer cells. Cancer Res. 2009;69(23):8902–8909. doi:10.1158/0008-5472.CAN-09-1404 19903851 PMC2805191

[51] Webster AC Ruster LP McGee R, . Interleukin 2 receptor antagonists for kidney transplant recipients. Cochrane Database Syst Rev. 2010;2010(1):CD003897. doi:10.1002/14651858.CD003897.pub3 20091551 PMC7154335

[52] Wimmer CD Rentsch M Crispin A, . The janus face of immunosuppression - de novo malignancy after renal transplantation: the experience of the Transplantation Center Munich. Kidney Int. 2007;71(12):1271–1278. doi:10.1038/sj.ki.5002154 17332737

[53] Kasiske BL Snyder JJ Gilbertson DT Wang C. Cancer after kidney transplantation in the United States. Am J Transplant. 2004;4(6):905–913. doi:10.1111/j.1600-6143.2004.00450.x 15147424

[54] Robson R Cecka JM Opelz G Budde M Sacks S. Prospective registry-based observational cohort study of the long-term risk of malignancies in renal transplant patients treated with mycophenolate mofetil. Am J Transplant. 2005;5(12):2954–2960. doi:10.1111/j.1600-6143.2005.01125.x 16303010

[55] Vincenti F Rostaing L Grinyo J, . Belatacept and long-term outcomes in kidney transplantation. N Engl J Med. 2016;374(4):333–343. doi:10.1056/NEJMoa1506027 26816011

[56] Durrbach A Pestana JM Pearson T, . A phase III study of belatacept versus cyclosporine in kidney transplants from extended criteria donors (BENEFIT-EXT study). Am J Transplant. 2010;10(3):547–557. doi:10.1111/j.1600-6143.2010.03016.x 20415898

[57] Masson P Henderson L Chapman JR Craig JC Webster AC. Belatacept for kidney transplant recipients. Cochrane Database Syst Rev. 2014;2014(11):CD010699. doi:10.1002/14651858.CD010699.pub2 25416857 PMC6486198

[58] Wang M Mittal A Colegio OR. Belatacept reduces skin cancer risk in kidney transplant recipients. J Am Acad Dermatol. 2020;82(4):996–998. doi:10.1016/j.jaad.2019.09.070 31589945

[59] Jew OS Liu WW Stamey C, . De novo belatacept does not reduce the rate of skin cancer in renal transplant recipients compared to standard therapy [published online ahead of print June 4, 2024]. J Am Acad Dermatol. doi:10.1016/j.jaad.2024.05.068 PMC11416314 38844009

[60] Dusendang JR Carlson E Lee DS, . Cohort and nested case-control study of cutaneous squamous cell carcinoma in solid organ transplant recipients, by medication. J Am Acad Dermatol. 2022;86(3):598–606. doi:10.1016/j.jaad.2021.07.065 34384835

[61] Dutcher JP Flippot R Fallah J Escudier B. On the shoulders of giants: the evolution of renal cell carcinoma treatment-cytokines, targeted therapy, and immunotherapy. Am Soc Clin Oncol Educ Book. 2020;40:1–18. doi:10.1200/EDBK_280817 32243201

[62] Lum EL Huang E Bunnapradist S Pham T Danovitch G. Acute kidney allograft rejection precipitated by lenalidomide treatment for multiple myeloma. Am J Kidney Dis. 2017;69(5):701–704. doi:10.1053/j.ajkd.2016.11.024 28189378

[63] Meyers DE Adu-Gyamfi B Segura AM, . Fatal cardiac and renal allograft rejection with lenalidomide therapy for light-chain amyloidosis. Am J Transplant. 2013;13(10):2730–2733. doi:10.1111/ajt.12391 23914832

[64] Garg N Rennke HG Pavlakis M Zandi-Nejad K. De novo thrombotic microangiopathy after kidney transplantation. Transpl Rev. 2018;32(1):58–68. doi:10.1016/j.trre.2017.10.001 29157988

[65] Moloney FJ Comber H O’Lorcain P O’Kelly P Conlon PJ Murphy GM. A population-based study of skin cancer incidence and prevalence in renal transplant recipients. Br J Dermatol. 2006;154(3):498–504. doi:10.1111/j.1365-2133.2005.07021.x 16445782

[66] Shao EX Betz-Stablein B Khosrotehrani K Campbell S Isbel N Green AC. Keratinocyte cancer mortality in kidney transplant recipients. Transplantation. 2022;106(5):1078–1083. doi:10.1097/TP.0000000000003827 34049361

[67] Ascha M Ascha MS Tanenbaum J Bordeaux JS. Risk factors for melanoma in renal transplant recipients. JAMA Dermatol. 2017;153(11):1130–1136. doi:10.1001/jamadermatol.2017.2291 28746700 PMC5710441

[68] Wisgerhof HC Edelbroek JRJ de Fijter JW, . Subsequent squamous- and basal-cell carcinomas in kidney-transplant recipients after the first skin cancer: cumulative incidence and risk factors. Transplantation. 2010;89(10):1231–1238. doi:10.1097/TP.0b013e3181d84cdc 20410852

[69] Euvrard S Morelon E Rostaing L, . Sirolimus and secondary skin-cancer prevention in kidney transplantation. N Engl J Med. 2012;367(4):329–339. doi:10.1056/NEJMoa1204166 22830463

[70] Yanik EL Siddiqui K Engels EA. Sirolimus effects on cancer incidence after kidney transplantation: a meta-analysis. Cancer Med. 2015;4(9):1448–1459. doi:10.1002/cam4.487 26108799 PMC4567030

[71] Duncan FJ Wulff BC Tober KL, . Clinically relevant immunosuppressants influence UVB-induced tumor size through effects on inflammation and angiogenesis. Am J Transplant. 2007;7(12):2693–2703. doi:10.1111/j.1600-6143.2007.02004.x 17941958

[72] Dantal J Morelon E Rostaing L, . Sirolimus for secondary prevention of skin cancer in kidney transplant recipients: 5-year results. J Clin Oncol. 2018;36(25):2612–2620. doi:10.1200/JCO.2017.76.6691 30016177

[73] Badve SV Pascoe EM Burke M, . Mammalian target of rapamycin inhibitors and clinical outcomes in adult kidney transplant recipients. Clin J Am Soc Nephrol. 2016;11(10):1845–1855. doi:10.2215/CJN.00190116 27445164 PMC5053777

[74] Coghill AE Johnson LG Berg D Resler AJ Leca N Madeleine MM. Immunosuppressive medications and squamous cell skin carcinoma: nested case-control study within the skin cancer after organ transplant (SCOT) cohort. Am J Transplant. 2016;16(2):565–573. doi:10.1111/ajt.13596 26824445 PMC5500236

[75] Murakami N Mulvaney P Danesh M, . A multi-center study on safety and efficacy of immune checkpoint inhibitors in cancer patients with kidney transplant. Kidney Int. 2021;100(1):196–205. doi:10.1016/j.kint.2020.12.015 33359528 PMC8222056

[76] Carroll RP Boyer M Gebski V, . Immune checkpoint inhibitors in kidney transplant recipients: a multicentre, single-arm, phase 1 study. Lancet Oncol. 2022;23(8):1078–1086. doi:10.1016/S1470-2045(22)00368-0 35809595

[77] Schenk KM Deutsch JS Chandra S, . Nivolumab + tacrolimus + prednisone ± ipilimumab for kidney transplant recipients with advanced cutaneous cancers. J Clin Oncol. 2024;42(9):1011–1020. doi:10.1200/JCO.23.01497 38252910 PMC11677297

[78] Hanna GJ Dharanesswaran H Giobbie-Hurder A, . Cemiplimab for kidney transplant recipients with advanced cutaneous squamous cell carcinoma. J Clin Oncol. 2024;42(9):1021–1030. doi:10.1200/JCO.23.01498 38252908 PMC10950183

[79] Van Meerhaeghe T Murakami N Le Moine A Brouard S Sprangers B Degauque N. Fine-tuning tumor- and allo-immunity: advances in the use of immune checkpoint inhibitors in kidney transplant recipients. Clin Kidney J. 2024;17(4):sfae061. doi:10.1093/ckj/sfae061 38606169 PMC11008728

[80] Sprangers B Riella LV Dierickx D. Posttransplant lymphoproliferative disorder following kidney transplantation: a review. Am J Kidney Dis. 2021;78(2):272–281. doi:10.1053/j.ajkd.2021.01.015 33774079

[81] Francis A Johnson DW Teixeira-Pinto A Craig JC Wong G. Incidence and predictors of post-transplant lymphoproliferative disease after kidney transplantation during adulthood and childhood: a registry study. Nephrol Dial Transplant. 2018;33(5):881–889. doi:10.1093/ndt/gfx356 29342279

[82] Kitchlu A Dixon S Dirk JS, . Elevated risk of cancer after solid organ transplant in childhood: a population-based cohort study. Transplantation. 2019;103(3):588–596. doi:10.1097/TP.0000000000002378 30048393

[83] van Leeuwen MT Grulich AE Webster AC, . Immunosuppression and other risk factors for early and late non-Hodgkin lymphoma after kidney transplantation. Blood. 2009;114(3):630–637. doi:10.1182/blood-2009-02-202507 19443660

[84] Opelz G Naujokat C Daniel V Terness P Döhler B. Disassociation between risk of graft loss and risk of non-Hodgkin lymphoma with induction agents in renal transplant recipients. Transplantation. 2006;81(9):1227–1233. doi:10.1097/01.tp.0000219817.18049.36 16699447

[85] Dharnidharka VR Ruzinova MB Marks LJ. Post-transplant lymphoproliferative disorders. Semin Nephrol. 2024;44(1):151503. doi:10.1016/j.semnephrol.2024.151503 38519279 PMC11213680

[86] Swerdlow SH Campo E Harris NL, . WHO Classification of Tumours of Haematopoietic and Lymphoid Tissues, 4th Edition, Volume 2. International Agency for Research on Cancer; 2017.

[87] Alaggio R Amador C Anagnostopoulos I, . The 5th edition of the world Health organization classification of haematolymphoid tumours: lymphoid neoplasms. Leukemia. 2022;36(7):1720–1748. doi:10.1038/s41375-022-01620-2 35732829 PMC9214472

[88] Reshef R Vardhanabhuti S Luskin MR, . Reduction of immunosuppression as initial therapy for posttransplantation lymphoproliferative disorder (★). Am J Transplant. 2011;11(2):336–347. doi:10.1111/j.1600-6143.2010.03387.x 21219573 PMC3079420

[89] Trappe R Hinrichs C Appel U, . Treatment of PTLD with rituximab and CHOP reduces the risk of renal graft impairment after reduction of immunosuppression. Am J Transplant. 2009;9(10):2331–2337. doi:10.1111/j.1600-6143.2009.02772.x 19663889

[90] Dierickx D Habermann TM. Post-transplantation lymphoproliferative disorders in adults. N Engl J Med. 2018;378(6):549–562. doi:10.1056/NEJMra1702693 29414277

[91] Neelapu SS Locke FL Bartlett NL, . Axicabtagene ciloleucel CAR T-cell therapy in refractory large B-cell lymphoma. N Engl J Med. 2017;377(26):2531–2544. doi:10.1056/NEJMoa1707447 29226797 PMC5882485

[92] Schuster SJ Bishop MR Tam CS, . Tisagenlecleucel in adult relapsed or refractory diffuse large B-cell lymphoma. N Engl J Med. 2019;380(1):45–56. doi:10.1056/NEJMoa1804980 30501490

[93] Krishnamoorthy S Ghobadi A Santos RD, . CAR-T therapy in solid organ transplant recipients with treatment refractory posttransplant lymphoproliferative disorder. Am J Transplant. 2021;21(2):809–814. doi:10.1111/ajt.16367 33089906

[94] Mamlouk O Nair R Iyer SP, . Safety of CAR T-cell therapy in kidney transplant recipients. Blood. 2021;137(18):2558–2562. doi:10.1182/blood.2020008759 33512386 PMC8109014

[95] Portuguese AJ Gauthier J Tykodi SS, . CD19 CAR-T therapy in solid organ transplant recipients: case report and systematic review. Bone Marrow Transplant. 2023;58(4):353–359. doi:10.1038/s41409-022-01907-z 36575360 PMC12977333

[96] Schreiber B Tripathi S Nikiforow S Chandraker A. Adoptive immune effector cell therapies in cancer and solid organ transplantation: a review. Semin Nephrol. 2024;44(1):151498. doi:10.1016/j.semnephrol.2024.151498 38555223

[97] McKenna M Epperla N Ghobadi A, . Real-world evidence of the safety and survival with CD19 CAR-T cell therapy for relapsed/refractory solid organ transplant-related PTLD. Br J Haematol. 2023;202(2):248–255. doi:10.1111/bjh.18828 37129856

[98] Fernandez-Cuesta L Perdomo S Avogbe PH, . Identification of circulating tumor DNA for the early detection of small-cell lung cancer. EBioMedicine. 2016;10:117–123. doi:10.1016/j.ebiom.2016.06.032 27377626 PMC5036515

[99] Aggarwal C Thompson JC Black TA, . Clinical implications of plasma-based genotyping with the delivery of personalized therapy in metastatic non-small cell lung cancer. JAMA Oncol. 2019;5(2):173–180. doi:10.1001/jamaoncol.2018.4305 30325992 PMC6396811

[100] Reinert T Henriksen TV Christensen E, . Analysis of plasma cell-free DNA by ultradeep sequencing in patients with stages I to III colorectal cancer. JAMA Oncol. 2019;5(8):1124–1131. doi:10.1001/jamaoncol.2019.0528 31070691 PMC6512280

[101] Fakih M Sandhu J Wang C, . Evaluation of comparative surveillance strategies of circulating tumor DNA, imaging, and carcinoembryonic antigen levels in patients with resected colorectal cancer. JAMA Netw Open. 2022;5(3):e221093. doi:10.1001/jamanetworkopen.2022.1093 35258578 PMC8905389

[102] Tie J Cohen JD Lahouel K, . Circulating tumor DNA analysis guiding adjuvant therapy in stage II colon cancer. N Engl J Med. 2022;386(24):2261–2272. doi:10.1056/NEJMoa2200075 35657320 PMC9701133

[103] Kumar S Paiva B Anderson KC, . International Myeloma Working Group consensus criteria for response and minimal residual disease assessment in multiple myeloma. Lancet Oncol. 2016;17(8):e328–e346. doi:10.1016/S1470-2045(16)30206-6 27511158

[104] Costa LJ Derman BA Bal S, . International harmonization in performing and reporting minimal residual disease assessment in multiple myeloma trials. Leukemia. 2021;35(1):18–30. doi:10.1038/s41375-020-01012-4 32778736

[105] Munshi NC Avet-Loiseau H Anderson KC, . A large meta-analysis establishes the role of MRD negativity in long-term survival outcomes in patients with multiple myeloma. Blood Adv. 2020;4(23):5988–5999. doi:10.1182/bloodadvances.2020002827 33284948 PMC7724898

[106] Dekker SE Rea D Cayuela JM Arnhardt I Leonard J Heuser M. Using measurable residual disease to optimize management of AML, ALL, and chronic myeloid leukemia. Am Soc Clin Oncol Educ Book. 2023;43:e390010. doi:10.1200/EDBK_390010 37311155

[107] Herrera AF Armand P. Minimal residual disease assessment in lymphoma: methods and applications. J Clin Oncol. 2017;35(34):3877–3887. doi:10.1200/JCO.2017.74.5281 28933999 PMC5707209

[108] Schroder PM Biesterveld BE Al-Adra DP. Premalignant lesions in the kidney transplant candidate. Semin Nephrol. 2024;44(1):151495. doi:10.1016/j.semnephrol.2024.151495 38490902

[109] Witherow BA Roth GS Carrozza MA, . The Israel Penn international transplant tumor registry. AMIA Annu Symp Proc. 2003;2003:1053. PMID: 14728556 14728556 PMC1480060

